# Gait Adaptation to a Phase-Specific Nociceptive Electrical Stimulation Applied at the Ankle: A Model to Study Musculoskeletal-Like Pain

**DOI:** 10.3389/fnhum.2021.762450

**Published:** 2021-12-17

**Authors:** Michaël Bertrand-Charette, Renaud Jeffrey-Gauthier, Jean-Sébastien Roy, Laurent J. Bouyer

**Affiliations:** ^1^Center for Interdisciplinary Research in Rehabilitation and Social Integration, Quebec City, QC, Canada; ^2^Department of Rehabilitation, Faculty of Medicine, Université Laval, Quebec City, QC, Canada

**Keywords:** pain, pain protocol, gait, adaptation, ankle, musculoskeletal

## Abstract

**Introduction:** Lower limb pain, whether induced experimentally or as a result of a musculoskeletal injury, can impair motor control, leading to gait adaptations such as increased muscle stiffness or modified load distribution around joints. These adaptations may initially reduce pain but can also lead to longer-term maladaptive plasticity and to the development of chronic pain. In humans, many current experimental musculoskeletal-like pain models are invasive, and most don’t accurately reproduce the movement-related characteristics of musculoskeletal pain. The main objective of this study was to measure pain adaptation strategies during gait of a musculoskeletal-like experimental pain protocol induced by phase-specific, non-invasive electrical stimulation.

**Methods:** Sixteen healthy participants walked on a treadmill at 4 km/h for three consecutive periods (BASELINE, PAIN, and POST-PAIN). Painful electrical stimulations were delivered at heel strike for the duration of heel contact (HC) using electrodes placed around the right lateral malleolus to mimic ankle sprains. Gait adaptations were quantified bilaterally using instrumented pressure-sensitive insoles. One-way ANOVAs and group time course analyses were performed to characterize the impact of electrical stimulation on heel and forefoot contact pressure and contact duration.

**Results:** During the first few painful strides, peak HC pressure decreased on the painful side (8.6 ± 1.0%, *p* < 0.0001) and increased on the non-stimulated side (11.9 ± 0.9%, *p* < 0.0001) while HC duration was significantly reduced bilaterally (painful: 12.1 ± 0.9%, *p* < 0.0001; non-stimulated: 4.8 ± 0.8%, *p* < 0.0001). No clinically meaningful modifications were observed for the forefoot. One minute after the onset of painful stimulation, perceived pain levels stabilized and peak HC pressure remained significantly decreased on the painful side, while the other gait adaptations returned to pre-stimulation values.

**Discussion:** These results demonstrate that a non-invasive, phase-specific pain can produce a stable painful gait pattern. Therefore, this protocol will be useful to study musculoskeletal pain locomotor adaptation strategies under controlled conditions.

## Introduction

In the presence of acute pain, whether induced experimentally or as a result of a musculoskeletal injury, various sensorimotor modifications are often present. They include proprioceptive deficits, altered patterns of neuromuscular activations and/or altered movement kinetics/kinematics (Sterling et al., 2001; Bank et al., 2013). For example, after an ankle sprain, increased knee valgus at heel contact (HC) and reduced hip extension at toe-off (TO) can be observed in the injured limb (Crosbie et al., 1999; Doherty et al., 2015; Punt et al., 2015). Effects on the non-injured joints are also reported, such as reductions in ankle plantar flexion at HC and TO (Doherty et al., 2015). Similarly, a decrease in motor performance can even be seen in both limbs, as shown with the Star Excursion Balance Test (Bertrand-Charette et al., 2020).

While some of these changes are associated with the injured anatomical structure and affect mechanical joint stability (Laskowski et al., 2000), others lead to the protection of the painful limb, and to immediate pain reduction. According to Hodges and Tucker (2011), the repeated use of “protective” pain-avoidance motor strategies, while beneficial in the short-term, can become detrimental in the longer-term, and lead to pain chronicization. Indeed, the presence of pain can modify muscle stiffness and/or muscle recruitment, thereby changing the way load is applied on articular surfaces and lead to early wear of the locomotor apparatus. Transforming an initial pain-avoidance motor strategy into a regular motor pattern therefore represents a form of maladaptive learning (Hodges and Tucker, 2011) that should be avoided to prevent chronic pain development (Henriksen et al., 2011).

As inadequate management of acute pain could potentially increase the risk of developing chronic pain (Sinatra, 2010), it is of the utmost importance to better understand the impact of acute pain on lower limb motor control. In order to assess this impact, a valid musculoskeletal (MSK) pain model to study pain adaptation strategies must induce lasting effects, not only immediate withdrawal effects. Unfortunately, many current acute MSK pain models are invasive [e.g., intramuscular injections of hypertonic saline or adenosine (Madeleine et al., 1999; Henriksen et al., 2011)] and most don’t accurately represent the movement-related (or phasic) nature of MSK pain. For example, hypertonic saline or adenosine injections and ischemic contractions have been described as producing a tonic, continuous pain (Stohler et al., 1996; Bennell et al., 2004) that can induce both local and referred (widespread) pain (Graven-Nielsen et al., 2003). Regarding the latter, MSK injuries such as ankle sprain tend to generate mainly local pain around the injury site (Dubin et al., 2011). To better represent this aspect, previous pain models, such as the steel beads model of Levins et al. (1998), were designed to generate localized pain in order to alter gait pattern and study gait adaptations. The reduction in single-limb support on the painful limb is similar to what Hodges and Tucker (2011) suggested, however, this protocol cannot control parameters such as pain timing, duration, or intensity.

Gallina et al. (2021) have recently proposed a pain protocol using low-frequency sinusoidal electrical stimuli and showed that this type of electrical stimulation can induce knee pain of constant intensity for 60 s. However, the stimulation used was continuous. A protocol using nociceptive electrical stimulation that would be phase-specific (i.e., having adjustable pain intensity and present only at an MSK-pain relevant moment of the gait cycle), and being described as an acute MSK-like pain, would be more ecological to study the effects of experimental acute pain on gait motor control. Therefore, to avoid some of these limitations, an experimental pain model using electrical stimulation was developed. Pain induced by electrical stimulation is non-invasive, can produce a pain sensation of adjustable intensity, has the potential of being focal to the site around electrode location, and can be triggered at a specific moment of the gait cycle (Duysens et al., 1992).

The main objective of this study was therefore to characterize the impact of a phase-specific, painful electrical stimulation on gait adaptations. As gait is a complex multi-articular movement, we decided to focus the analysis of this study on two functionally important movement outputs during gait: the HC phase representing the initial contact and weight acceptance phases of the gait cycle, and push-off, a key part of the pre-swing phase associated with the control of gait speed (Dean et al., 2000). Vertical force magnitude and support duration were measured in these two regions of interest (ROIs) using pressure-sensitive insoles. As pain perception can be quite diverse, the secondary objective of this study was therefore to qualify the nociceptive stimulus perceived by the participants in order to highlights the potential similarities between “MSK” aspects of a real acute pain and the actual electrical nociceptive stimulation delivered.

Our main hypothesis was that electrically evoked phasic pain (and not a non-painful stimulation) would modify the gait into a pain-avoidance strategy, leading to a gait pattern modification beyond an initial pain-avoidance strategy (as reported clinically; O’Connor et al., 2013). This would therefore be a good experimental model to later study MSK-like pain during gait. In addition, we hypothesized that the pain generated would have similar qualities to acute MSK-like pain, i.e., local at the application site, phase-specific and qualified mainly by sensory pain descriptors, such as those reported in the Short-Form McGill Pain Questionnaire-2 (SF-MPQ-2).

## Materials and Methods

### Participants

A convenience sample of 16 young healthy participants (28.2 ± 4.8 years old; 8 females) was recruited from *Université Laval* student population for this single-day, repeated measures design study. Participants had to be naïve to the task and present no self-reported pain. The exclusion criteria were self-reported symptoms or movement limitations at the lower limb or any neurological impairment that could affect task performance. All participants read and signed a consent form describing the experimental procedure and their involvement in the study. This protocol was approved by the local Ethics Review Board (CIUSSS-CN, #2010-212). The experimental procedures were in accordance with the Declaration of Helsinki.

### General Protocol

Participants took part in a 2-h laboratory session. After filling the Waterloo Footedness Questionnaire (WFQ; Elias et al., 1998), they walked at 4 km/h on a motorized treadmill (Biodex Gait Trainer 2) for four periods: a 5-min PRE-BASELINE period to familiarize with treadmill walking and to set individual painful stimulation intensity, a 3-min BASELINE period, where they walked without any stimulation, a 3-min PAIN period with stimulation on every gait cycle, and a 3-min POST-PAIN period with no stimulation. Short rest moments (<30 s) were given between the four walking periods. Participants wore shoes instrumented with pressure-sensitive insoles (Tekscan F-Scan, South Boston, MA, United States) to collect dynamic pressure distribution under the foot and temporal gait parameters. They were instructed to walk on the treadmill normally, and to keep walking as they would normally in the presence of pain.

During the PAIN period, they verbally rated ankle pain intensity every 15 s using a numeric Visual Analog Scale (VAS; range 0–10). Immediately after the PAIN period, they rated their global unpleasantness intensity using a modified numeric VAS incorporating the anchors “not bad at all” and “the most unpleasant imaginable” (Duncan et al., 1989), painful region size by pointing at circles of different diameters (Bennell et al., 2004), and pain location by pointing on a schematic shank and foot chart (Bennell et al., 2004). Upon completion of the treadmill walking test, participants completed the SF-MPQ-2. The SF-MPQ-2 consists of 22 pain descriptors divided into four sub-scales: continuous pain, intermittent pain, predominantly neuropathic pain, and affective (Dworkin et al., 2015).

### Painful Electrical Stimulation

**FIGURE 1:**
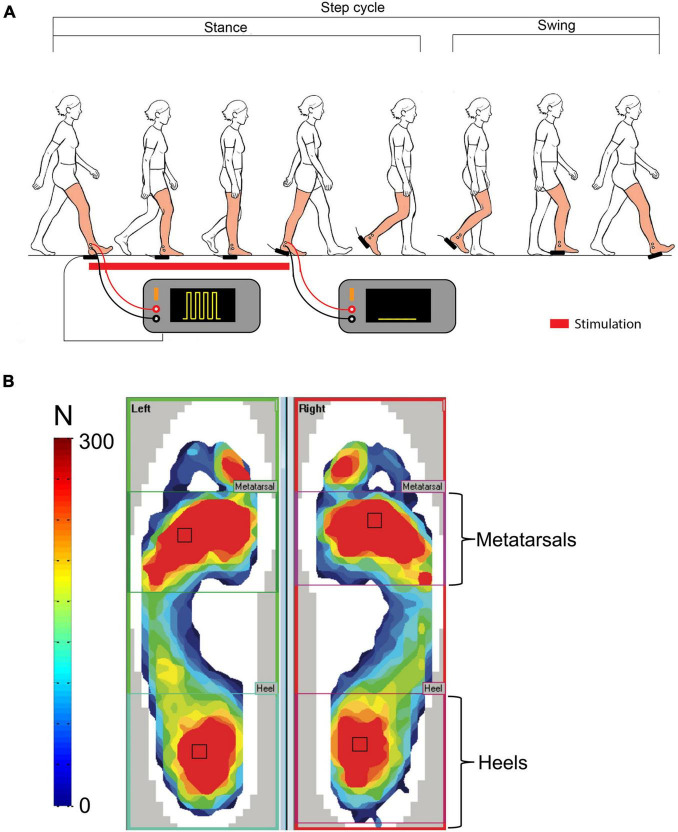
Regions of interest. **(A)** Schematic representation of the pain-generating set-up and electrical stimulus waveform. **(B)** Representation of the two functional regions of interest: heels and metatarsals.

Real-time heel-contact duration was measured using a pressure-sensitive foot switch located under the right heel. It served as stimulus trigger and stimulus duration control. There was no time delay between HC and actual electrical stimulus onset. A home-made electronic circuit interfaced the foot switch with two Grass s-88 stimulators (Grass Instruments, Quincy, MA, United States) wired in-series for signal generation, and a Digitimer DS7A stimulator (Hertfordshire, United Kingdom) for stimulus delivery to the participants. The painful electrical stimulation consisted of series of five pulses (pulse width: 500 μs; pulse frequency: 200 Hz) delivered in bursts at 30 Hz, for the duration of individual right HCs (Figure 1A). Kendall 2.2 cm^2^ H69P disposable electrodes were placed on the right lateral malleolus and 2 cm further along the distal end of the fibula and used for stimulus delivery. Intensity required to reach 3/10 on the VAS was determined for each participant during the PRE-BASELINE period (steps of 5 mA increased every 10 s) and maintained constant throughout the experiment.

### Non-painful Electrical Stimulation Controls

In a subgroup of five participants, a second walking test was performed on a separate day in the presence of non-painful stimulation, to assess the contribution of stimulation distraction on the gait biomechanical parameters. Walking periods duration, order, etc., remained the same except for stimulation intensity that was set at 1.4× perceptual threshold (PT), compared to approximately 3.0× PT for the pain experiment.

### Gait Adaptations Characterization

F-Scan pressure-sensitive insoles (Tekscan, South Boston, MA, United States) were used to collect dynamic pressure distribution under the foot and temporal gait parameters. Peak foot pressure magnitude and duration in the heel and metatarsal regions (see section “Materials and Methods” and Figure 1B) were quantified for each stride of the BASELINE, PAIN, and POST-PAIN walking periods, bilaterally.

### Data Analysis

Stride-to-stride duration of the nociceptive electrical stimulation was measured off-line from recordings of the pulse trains using a custom-made program written in MATLAB (Version R2018b, MathWorks, United States). The Tekscan data analysis software (F-Scan Research V7.5; Tekscan, United States) was used to set the ROIs on the pressure data from the insoles around the heels and the metatarsals for each participant (Figure 1B). These two ROIs were selected as they represent two functionally important outputs of movement strategies during gait: HC representing the initial contact at heel strike, weight acceptance and mid-stance phases of the gait cycle, and push-off a key part of the pre-swing phase associated with gait speed (Dean et al., 2000). These functional ROIs will be referred to as heel and metatarsal regions throughout this paper, respectively. Both peak pressure magnitude and contact duration of these ROIs were extracted and analyzed with a custom-made MATLAB program.

### Statistics

To measure the effects of pain on gait adaptations, two complementary analyses were performed; (1) evolution over time for the group data (Fortin et al., 2009; Bertrand-Charette et al., 2021) of the time course (Cumming and Finch, 2005); (2) multi-epochs (see below) for statistical comparisons (see Bertrand-Charette et al., for details).

#### Multi-Epochs Analysis

The following epochs were defined:

(1) BASELINE late: mean of the last 50 strides of the group BASELINE period;
(2) PAIN early: mean of the first 5 strides of the group PAIN period;
(3) PAIN late: mean of the last 50 strides of the group PAIN period;
(4) POST-PAIN early: mean of the first 5 strides of the group POST-PAIN period;

One-way ANOVA with Dunnett’s multiple comparisons test were performed to compare these epochs using GraphPad Prism (version 9.0.0). The level of significance was set at 0.05.

#### Group Time Course Analysis

A 95% confidence interval (CI_95%_) was calculated from the last 50 baseline strides to represent normal stride-to-stride variability observed during BASELINE walking. PAIN and POST-PAIN data were then compared to this CI_95%_ using an 11-points moving average line as a visual reference. Moving average values outside of the CI_95%_ were considered as significantly different from baseline.

#### Questionnaires

Individual scores on the WFQ and SF-MPQ-2 were extracted and pooled to report the footedness and frequency of perceived pain qualities.

#### General Unpleasantness, Painful Area Size, and Location

Data collected for each participant were extracted and pooled to report the group painful area size and location on the ankle.

#### Duration of the Nociceptive Stimulation

The duration of each stimulation was extracted for each participant and pooled to report mean duration and standard deviation.

## Results

### Participants’ Characteristics

The group was composed of 16 participants (mean age: 28.2 ± 4.8 years; 8 females; see Table 1 for participants’ characteristics).

**TABLE 1:** Participants’ characteristics.

Characteristics	
Age	28.2 ± 4.8
Sex	
• Male	8
• Female	8
Footedness	
• Right	14
• Left	2
Stimulation intensity (mA)	14.4 ± 5.2
Mean VAS score (range 0-10)	2.5 ± 0.1
Unpleasantness score (range 0-10)	4.3 ± 1.7

*mA, milliamps; VAS, Visual Analog Scale.*

### Stimulus Intensity, Duration, and Pain Level During Gait

**FIGURE 2:**
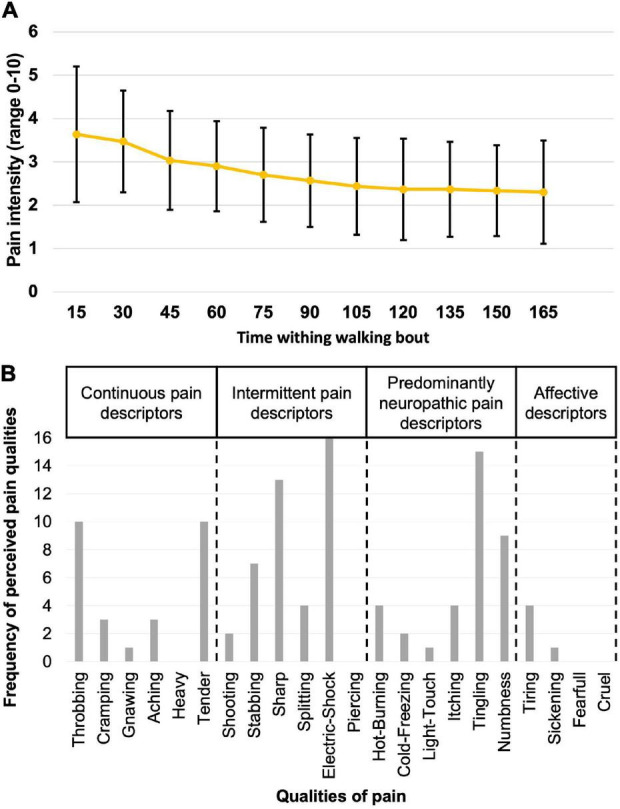
Description of the perceived pain. **(A)** The mean score and standard deviation (black lines) on the Visual Analog Scale for pain intensity as measured every 15 s for all participants while walking during the PAIN period. **(B)** Frequency of perceived pain qualities, as assessed using the Short-Form McGill Pain Questionnaire-2.

**FIGURE 3:**
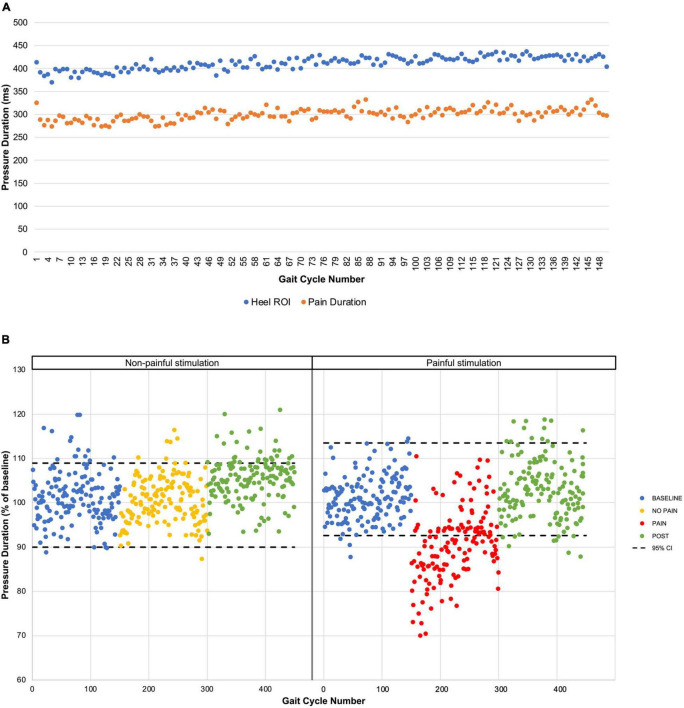
Pressure duration time course. **(A)** The mean pressure durations for each gait cycle during PAIN period are presented for the heel ROI extracted from the pressure-sensitive insoles (blue dots) and for the pressure-sensitive foot switch located under the right heel (orange dots). **(B)** Results from the non-painful stimulation control experiment. Mean pressure durations are presented for the five participants for each period with the 95% confidence interval (black dashed line) based on the BASELINE.

The mean stimulus intensity necessary to obtain a phasic painful stimulation of 3/10 on the VAS during gait at the onset of the pain period was 14.4 ± 5.2 mA. While stimulus intensity was maintained fixed throughout the PAIN period, pain intensity perceived by the participants slightly decreased over the first 60 s, and then stabilized at 2.5 ± 0.4/10 (time course shown in Figure 2A). Pain tended to be centered on the right lateral malleolus with a mean diameter of 3.3 ± 1.3 cm. No radiating pain was reported by participants. The mean duration of the nociceptive stimulation was 300.7 ± 59 ms, 112.1 ± 12.1 ms shorter than the total HC phase measured with the insoles (Figure 3A).

### Perceived Pain Qualities of the Nociceptive Electrical Stimulation

Participants rated the general unpleasantness of the stimulation session at 4.3 ± 1.7/10. According to the results of their SF-MPQ-2 (Figure 2B), the electrical stimulus was described as throbbing (*n* = 10/16), sharp (*n* = 13/16), tender (*n* = 10/16), electric-shock (*n* = 16/16), and tingling (*n* = 15/16). Other qualities of pain reported were stabbing and numbness (*n* = 7/16). All perceived qualities of pain are presented in Figure 2B.

### Effect of Painful Electrical Stimulation on Gait Adaptations

As mentioned in section “Materials and Methods,” gait is a complex multi-articular movement resulting in various movement strategies. Therefore, this section will present our results according to the two functionally important movement outputs identified by our ROIs (heel for weight acceptance; metatarsals for push-off), separately for peak pressure and contact duration.

#### Epoch Analysis for Peak Pressure Magnitude

Regarding the heel ROIs, on the stimulated side, HC peak pressure magnitude was significantly reduced by 8.6 ± 1.0% (*p* < 0.0001) and 8.1 ± 0.4% (*p* < 0.0001) during the PAIN early and PAIN late, respectively, compared to BASELINE late. During the PAIN early, on the non-stimulated side, HC peak pressure magnitude significantly increased by 11.9 ± 0.9% (*p* < 0.0001). For the metatarsal ROIs, significant changes can be found on the stimulated side for PAIN early (1.9 ± 0.7% reduction; *p* < 0.05), PAIN late (2.3 ± 0.3% increase; *p* < 0.0001), and POST-PAIN early (3.8 ± 0.7% reduction; *p* < 0.0001) compared to BASELINE late. On the non-stimulated side, a significant reduction of 5.5 ± 0.7% (*p* < 0.0001) can be observed during PAIN early when compared to BASELINE late. Overall, 10 participants reduced their HC peak pressure magnitude on the stimulated side during the PAIN early period, while 9 of them increased their HC peak pressure magnitude on the non-stimulated side. There was no relationship between pain intensity and HC peak pressure magnitude (see Supplementary Figure 1).

#### Epoch Analysis for Contact Duration

Regarding HC duration, a significant reduction was observed during PAIN early (mean duration reduction of 12.1 ± 0.9%; *p* < 0.0001) and PAIN late (mean duration reduction of 4.4 ± 0.4%; *p* < 0.0001) while an increase can be observed in POST-PAIN early (mean duration increase of 3.4 ± 0.9%; *p* < 0.001) for the right heel ROI. For the non-stimulated side, a mean reduction of 4.8 ± 0.8% (*p* < 0.0001) was present for the PAIN early epoch. For the metatarsal ROIs, a significant mean reduction of 9.3 ± 1.4% (PAIN early, *p* < 0.0001) was present on the stimulated side, but nothing during PAIN late and POST-PAIN early. On the non-stimulated side, a significant 2.7 ± 0.6% (*p* < 0.0001) reduction can be observed for PAIN early only. There was no relationship between pain intensity and contact duration. Overall, HC duration was reduced for 12 participants on the stimulated side and 11 participants on the non-stimulated side during PAIN early.

#### Peak Pressure Magnitude Time Course

**FIGURE 4:**
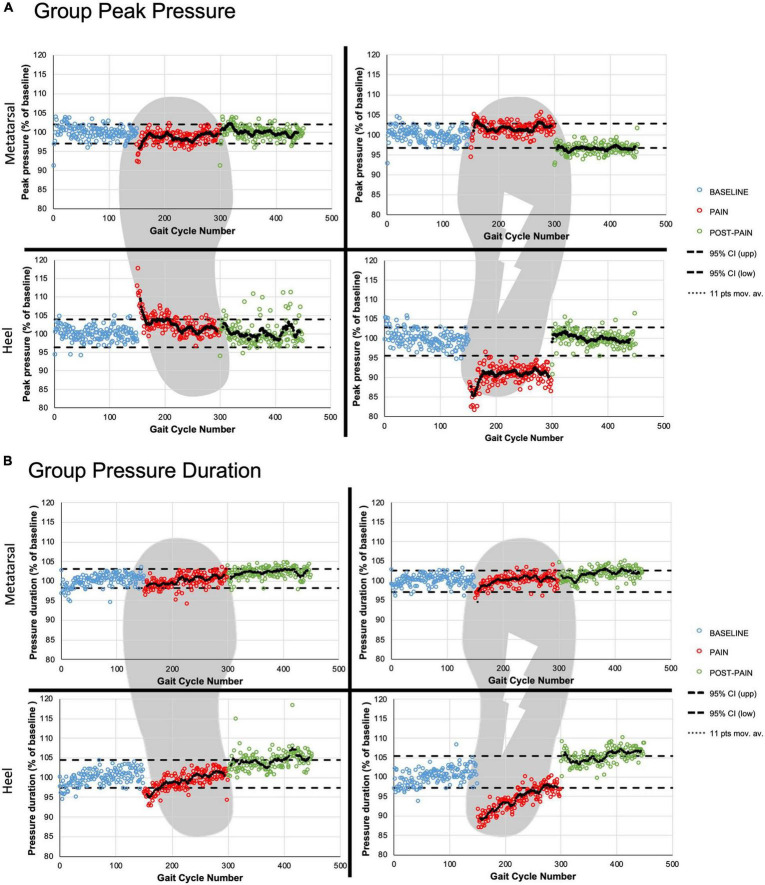
Group time courses. The three periods are presented for group peak pressure magnitude **(A)** and group contact duration **(B)** with the 95% confidence interval (black dashed line) based on the BASELINE. The two ROIs are presented for the right (painful) and the left (non-stimulated) foot. The black dotted lines represent the 11-points moving average for the PAIN and POST-PAIN periods.

A statistically significant drop in peak pressure during HC was observed for the PAIN period on the stimulated side only (Figure 4A). Peak HC pressure decreased to a mean 90.6 ± 4.0% of baseline value for the first five strides (PAIN early, *p* < 0.0001) and stabilized under the lower CI_95%_ of BASELINE for the remaining of the painful period.

In contrast, the non-stimulated side had a significant increase in HC peak pressure magnitude, that can be seen at the beginning of the painful period (mean maximal increase of 12.0 ± 3.8% of baseline values for PAIN early, *p* < 0.0001). Thereafter, the HC peak pressure moving average line remained around the upper CI_95%_ for the rest of PAIN period.

For the metatarsal ROIs, the moving average line remained most of the time within the CI_95%_ for both pain periods: less than 25 strides were over the upper CI_95%_ for the painful side, and below the lower CI_95%_ for the non-stimulated side.

For the heel ROIs during POST-PAIN period, participants returned to their normal HC peak pressure. Only the right metatarsal ROI showed peak pressure values around the lower CI_95%_ (POST-PAIN late: 96.8 ± 1.7% vs. lower CI_95%_: 96.7%).

#### Contact Duration Time Course

Regarding HC duration on the painful side, the moving average line was below the CI_95%_ for most of the painful period (Figure 4B). Mean duration spent on the right heel for PAIN early was 391.0 ± 7.2 ms compared to 443.8 ± 8.9 ms during BASELINE late value (*p* < 0.0001), showing a 12.1 ± 0.9% reduction. When looking at the non-stimulated side, a transient significant reduction in contact duration was observed for the first 43 steps of the PAIN period when compared to the baseline HC duration.

For the metatarsal ROIs, the moving average lines remained within the CI_95%_ most of the time during PAIN.

Regarding the POST-PAIN period, all ROIs values tend to vary around to the upper CI_95%_, with the right metatarsal ROI being the only ROI significantly increased compared to baseline values. This increase represents a 3.4 ± 0.9% (*p* < 0.001) of BASELINE late.

### Non-painful Stimulation Control Experiment

The effect of non-painful electrical stimulation on heel-contact duration was also tested in a subgroup of five participants on a different day. Results show that the non-painful stimulation did not affect HC duration (see Figure 3B).

## Discussion

The present study demonstrates that a painful phasic electrical stimulation applied at the ankle can modify the gait pattern beyond an initial pain-avoidance response in healthy participants. By assessing peak pressure magnitude and contact duration of the heel and metatarsal ROIs bilaterally as a means of quantifying the presence of gait adaptations, our results suggest that this protocol can recreate pain-avoidance reactions during the first few strides for duration and peak pressure magnitude. Moreover, the results suggest that this painful stimulation generates a modified painful gait pattern with a persistent reduction in HC peak pressure magnitude for the remaining of pain exposure. As a control, five of the participants returned to the lab for a similar experiment, in the presence of non-nociceptive stimulation. No change in contact duration was observed during the non-nociceptive stimulation, suggesting that the effect seen here is pain-specific.

### Gait Adaptations With Electrical Pain

Participants modified their gait pattern in the presence of phasic nociceptive electrical stimulation. It is important to notice that not only did they modify their gait (HC duration and peak pressure) during the initial phase of the PAIN period (PAIN early), as hypothesized, but they also showed a persisting reduction of their HC peak pressure magnitude that stabilized for the rest of the stimulated period (PAIN late). These two periods will be discussed separately below.

During PAIN early, there was a significant reduction in HC peak pressure and HC contact duration, indicative of an unloading of the painful limb. Even though the stimulation protocol was not designed to generate a complete withdrawal response on the stimulated side (withdrawal caused by higher stimulus intensities such as those presented in Spaich et al. (2004), this unloading suggests that stimulation was painful enough to alter the gait pattern. This effect on the painful side was associated with an increased HC peak pressure on the non-stimulated side, indicative of a dynamic increase in weight transfer to that limb (loading). This bilateral change in motor strategy could be interpreted as a protective response, based on Hodges and Tucker (2011).

After approximatively 25 strides into the PAIN period, HC peak pressure stabilized below the lower CI_95%_, i.e., at a significantly lower level than before PAIN period. This pressure level was then maintained stable for the rest of the pain period, i.e., for approximately 125 more strides (Figure 4A). Participants therefore showed a persisting reduction in HC peak pressure on the painful side. HC contact duration continued to tend toward baseline values until the end of the pain period. On the non-stimulated side, both pressure magnitude and contact duration returned to BASELINE values. Together these results suggest that the electrical nociceptive stimulus not only generated acute pain-avoidance gait modifications (as mentioned above), but also lead to the development of a persistent modification in gait pattern for the duration of the pain exposure. It is this modified gait pattern that will enable further studies on sensorimotor control or gait modifications with a well-controlled nociceptive stimulus in the future. The current protocol therefore brings a simple and powerful tool to further our knowledge in this field of research.

During the POST-PAIN period, statistically significant changes were observed, mainly for HC duration on the stimulated side. However, these changes were fairly small compared to the main effect observed during the PAIN period. As an example, on the painful side, an increase of 3.4 ± 0.9% relative to baseline duration is measured, compared to the 12.1 ± 0.9% decrease observed for PAIN early. Such small changes are therefore unlikely to be functionally or clinically meaningful.

### Link Between Heel Contact Pressure Duration and Stimulation Duration

Even if participants modified their contact duration to modulate pain duration (similar to what has been seen in Gallina et al. (2021) with task-relevant modulation of perceived pain intensity), they still maintained their unloading of the painful limb (reduced HC peak pressure) to possibly continue to “protect” the painful limb. We suggest that this persistent behavior could represent a maladaptive gait modification, according to Hodges and Tucker (2011). Using nociceptive electrical stimulation is therefore a powerful pain model that mimics possible adaptation to modulate pain as shown in Gallina et al. (2021) and by our results.

### Perceived Qualities of the Nociceptive Stimulus

Regarding painful area size and location, the electrical stimulation used in our protocol was localized around the stimulation site (3.3 ± 1.3 cm), similar to the result of Gallina et al. (2021). This is a major improvement compared to saline or adenosine injections and ischemic block (Graven-Nielsen et al., 2003), with participants reporting pain in various location below their knee.

As a means of further describing the qualities of the pain generated, previous studies using other experimental pain models simulating lower limb MSK pain (saline injections, ischemic block) have used the McGill Pain Questionnaire (versions 1 or 2). They have reported various qualities from the sensory subgroups of the SF-MPQ-2 including *Aching* and *Throbbing* (Bennell et al., 2004; Smith et al., 2020) or *Stabbing*, *Cramping*, *Burning*, *Heavy*, and *Exhausting* (Graven-Nielsen et al., 2003).

In the present study, *Throbbing*, *Sharp*, *Tender*, *Electric-shock*, and *Tingling* were the most frequently reported pain qualities. *Sharp* and *Electric-shock* have been categorized as *intermittent* pain descriptors, *Throbbing* and *Tender* as *continuous*, and *Tingling* as *predominantly neuropathic* (Dworkin et al., 2009). These subcategories were suggested by Dworkin et al. (2009, 2015), for participants suffering from chronic pain and acute low back pain. It has been shown that people suffering from acute pain tend to use sensory subgroups (continuous, intermittent, and predominantly neuropathic descriptors) more frequently compared to participants with chronic pain, that tend to score more frequently the affective descriptors (Reading, 1982). Due to the electrical nature of the pain used in our protocol, it is not surprising to have a higher number of participants reporting *Electric-shock* and *Tingling* over *Burning* or *Cramping* (frequent with the other models). However, in the present study, participants mainly reported sensory subgroups pain descriptors, and only rarely affective ones. This further supports the fact that the electrical nociceptive stimulation represents a good acute pain model.

### Comparisons to Other Pain Models

#### Similarities in Term of Motor Response to Pain

The electrically evoked phasic ankle pain protocol led to effects similar to those of other pain models used to study gait modifications. Regarding HC peak pressure reduction on the painful side, Madeleine et al. (1999) noted similar modifications following saline injections in the tibialis anterior, where participants tended to put less weight on the injected leg. Also, Seeley et al. (2013) noticed a decrease in peak vertical impact ground reaction force of 3–4% following injection in the infrapatellar fat pad. Regarding the decrease in HC duration, Levins et al. (1998) also noted a decrease in single limb support duration while using steel beads under the heel to create the painful stimulus. Such similarities with other experimental pain models further support the validity of our electrically evoked phasic ankle pain model during gait. One main difference, however, is that injections create a tonic continuous pain that rapidly increases and then gradually reduces (Madeleine et al., 1999; Graven-Nielsen et al., 2003). On the contrary, painful electrical stimulation can be triggered at a specific moment of the gait cycle as shown in this study and the pain intensity can be modulated by participants (Gallina et al., 2021). Our model therefore leads to the same effect observed throughout their experiment. However, since time-courses aren’t available to compare their results to ours, it is difficult to further conclude in terms of initial vs. late effects.

Previous work with electrical stimulation showed some similarities with our phase-specific ankle pain model. Studies investigating painful electrical stimulation at the lower back (Moseley et al., 2004; Moseley and Hodges, 2005) and at the knee (Tucker et al., 2012; Gallina et al., 2021) showed an altered motor response in the assessed muscles. Even if these studies used movement to trigger pain [for example arm movement to elicit painful stimulation of the lower back (Moseley and Hodges, 2005) or shifting body weight to modulate pain perception at the knee (Gallina et al., 2021)], none of them used a functional activity such as walking to trigger a painful stimulation. This phase-specific aspect of our pain model made it possible to study how pain can alter motor response during a task involving sensory gating and sensorimotor processing (Nielsen, 2003).

#### Advantages of the Electrically-Evoked *Phasic* Pain

In addition to the similarities with previous experimental pain models, using electrical stimulation allowed us to generate a local, phase-specific, non-invasive pain. Importantly, the mild to moderate pain level of 3–4/10 on the VAS (Boonstra et al., 2014) was easily reached for all participants at relatively low stimulation intensities (maximal intensity: 27 mA). Furthermore, this corresponds to what is typically reported during gait following grade I or II lateral ankle sprains (LASs) (Ivins, 2006). Electrode placement alongside the distal end of the lateral malleolus evoked a localized pain while avoiding radiating pain toward the foot. This is an improvement over other pain models, such as saline injections or ischemic block (Graven-Nielsen et al., 2003), where radiating pain was reported to a larger region than targeted. Only one of our participants felt pain from the lateral malleolus to the lateral side of the calcaneum. Moreover, electrical stimulation is less invasive and no flares are present hours after initial exposure to the stimulus (Petersen and Rowbotham, 1999).

A major advantage of this painful stimulation protocol is that it can be adjusted in its timing of application to target a specific moment in the gait cycle, i.e., it is phase-specific. Unlike other pain models such as capsaicin, saline, or ischemic block, which are described as a tonic continuous pain (Graven-Nielsen et al., 2003; Bouffard et al., 2014), the painful stimulus used in this study was present only during HC and lasted less than 500 ms.

This phasic aspect is closer to what is experienced during actual MSK pain. Levins et al. (1998) used steel beads to create a phasic plantar heel pain experimentally. Similar to our results, Levins et al. (1998) reported that participants reduced the amount of time spent on the painful limb. However, unlike the electrical stimulation proposed here, they could not precisely set the pain timing, duration, or intensity, as it could vary across participants depending on their mass, gait speed, and gait pattern (initial and adapted). The electrically evoked phasic ankle pain protocol presented in this study also shows direct similarities with actual ankle injuries. Regarding HC peak pressure, Doherty et al. (2015) suggested that, following a first acute ankle sprain, patients tend to use a “*compensatory mechanism*” that consists of attenuating impact forces at HC. Similar results can be seen during other movements, such as a drop vertical jump, where participants with an acute ankle sprain are offloading the injured limb or increasing the load on the non-injured limb (Doherty et al., 2014).

### Strengths and Limitations of the Study

This study has some limitations. First, the relatively young adult group and relatively small sample size that was recruited might limit the generalizability of the results. Also, participants walked on a treadmill, which may not be as functional as walking overground, but was necessary for our stimulation setup. Regarding the pain intensity, following the first minute of pain exposure, the perceived pain level stabilized around 2.5/10 (compared to the 3/10 initially reached). This could be partly explained by the electrical nature and parameters of the stimulation, close to what is used for transcutaneous electrical nerve stimulation (TENS), known for its hypoalgesic effects in healthy participants (Chesterton et al., 2002, 2003). Using monophasic square waveform at 300 Hz with an intensity of 14.4 ± 5.2 mA [compared to the 7.4 ± 2.2 mA sinusoidal waveform at 4 Hz in Gallina et al. (2021)] show similarities to usual TENS parameters (Chesterton et al., 2003). Importantly, this 0.5-point reduction in pain score had no major impact on the objectives of the study, that were to study gait modifications in the presence of pain. Now knowing that these stimulation parameters can induce changes in pain intensity, it will be possible to conduct future studies using parameters similar to Gallina et al. (2021). In addition, adjusting pain intensity to reflect the amount of pressure on the heel could be an improvement to even better represent the MSK-like aspect of our protocol. Finally, collecting electromyographic data would’ve made it possible to determine if flexion reflex were elicited following painful stimulation.

This study also has several strengths. It presents an original protocol to elicit pain experimentally that shares characteristics similar to actual MSK pain, in order to study human adaptation to nociceptive stimulation. One of the highlights of this phase-specific pain model is its easily adjustable nature (in terms of phase, duration, and intensity at any moment of the experiment), that allowed being present at a functionally relevant moment of the gait cycle. Another highlight is the longer-lasting gait modifications that quickly stabilized to obtain a robust modified gait pattern during the painful condition. Moreover, this model is non-invasive making it safe and easy to use in many settings and populations. Finally, it is possible to recreate gait adaptations that are found in other validated pain models and actual MSK injuries.

## Conclusion

These results support the use of the proposed phase-specific electrically evoked phasic ankle pain protocol to study gait adaptations in the presence of MSK-like pain. This protocol is an attractive MSK-like pain model, as it is non-invasive and can target specific, functionally relevant moments of the gait cycle, and shows similarities with actual MSK pain adaptation strategies. Future studies will use this protocol to further investigate the similarities of persisting gait adaptations to those observed during actual MSK pain, and thereby advance our understanding of the effects of MSK pain on global motor control.

## Data Availability Statement

The original contributions presented in the study are included in the article/Supplementary Material, further inquiries can be directed to the corresponding author.

## Ethics Statement

The studies involving human participants were reviewed and approved by the Centre Intégré Universitaire de Santé et de Services Sociaux de la Capitale-Nationale Ethics Review Board. The patients/participants provided their written informed consent to participate in this study.

## Author Contributions

MB-C, J-SR, and LJB contributed to study conception and design. MB-C, RJ-G, and LJB conducted the data collection and performed the data validation and analysis. MB-C wrote the draft of the manuscript and prepared the figures. All authors provided substantive feedback on the manuscript, contributed to the final manuscript, and read and approved the final manuscript.

## Conflict of Interest

The authors declare that the research was conducted in the absence of any commercial or financial relationships that could be construed as a potential conflict of interest.

## Publisher’s Note

All claims expressed in this article are solely those of the authors and do not necessarily represent those of their affiliated organizations, or those of the publisher, the editors and the reviewers. Any product that may be evaluated in this article, or claim that may be made by its manufacturer, is not guaranteed or endorsed by the publisher.

## Supplementary Material

Supplementary Figure 1 Individual time courses for the pain intensity (red line), pressure duration (pressure-sensitive insoles; blue line), and foot switch located under the right heel (orange line) for each participant.

## References

[B1] Bank P. J. Peper C. E. Marinus J. Beek P. J. van Hilten J. J. (2013). Motor consequences of experimentally induced limb pain: a systematic review. *Eur. J. Pain* 17 145–157. 10.1002/j.1532-2149.2012.00186.x 22718534

[B2] Bennell K. Hodges P. Mellor R. Bexander C. Souvlis T. (2004). The nature of anterior knee pain following injection of hypertonic saline into the infrapatellar fat pad. *J. Orthop. Res.* 22 116–121. 10.1016/S0736-0266(03)00162-1 14656669

[B3] Bertrand-Charette M. Dambreville C. Bouyer L. J. Roy J.-S. (2020). Systematic review of motor control and somatosensation assessment tests for the ankle. *BMJ Open Sport Exerc. Med.* 6:e000685. 10.1136/bmjsem-2019-000685 32655878 PMC7342858

[B4] Bertrand-Charette M. Nielsen J. B. Bouyer L. J. (2021). A simple, clinically applicable motor learning protocol to increase push-off during gait: a proof-of-concept. *PLoS One* 16:e0245523. 10.1371/journal.pone.0245523 33465113 PMC7815130

[B5] Boonstra A. M. Schiphorst Preuper H. R. Balk G. A. Stewart R. E. (2014). Cut-off points for mild, moderate, and severe pain on the visual analogue scale for pain in patients with chronic musculoskeletal pain. *Pain* 155 2545–2550. 10.1016/j.pain.2014.09.014 25239073

[B6] Bouffard J. Bouyer L. J. Roy J.-S. Mercier C. (2014). Tonic pain experienced during locomotor training impairs retention despite normal performance during acquisition. *J. Neurosci.* 34:9190. 10.1523/JNEUROSCI.5303-13.2014 25009252 PMC4087202

[B7] Chesterton L. S. Barlas P. Foster N. E. Lundeberg T. Wright C. C. Baxter D. G. (2002). Sensory stimulation (TENS): effects of parameter manipulation on mechanical pain thresholds in healthy human subjects. *Pain* 99 253–262. 10.1016/s0304-3959(02)00118-5 12237203

[B8] Chesterton L. S. Foster N. E. Wright C. C. Baxter D. G. Barlas P. (2003). Effects of TENS frequency, intensity and stimulation site parameter manipulation on pressure pain thresholds in healthy human subjects. *Pain* 106 73–80. 10.1016/s0304-3959(03)00292-6 14581113

[B9] Crosbie J. Green T. Refshauge K. (1999). Effects of reduced ankle dorsiflexion following lateral ligament sprain on temporal and spatial gait parameters. *Gait Posture* 9 167–172. 10.1016/s0966-6362(99)00010-7 10575077

[B10] Cumming G. Finch S. (2005). Inference by eye: confidence intervals and how to read pictures of data. *Am. Psychol.* 60:170. 10.1037/0003-066x.60.2.170 15740449

[B11] Dean C. M. Richards C. L. Malouin F. (2000). Task-related circuit training improves performance of locomotor tasks in chronic stroke: a randomized, controlled pilot trial. *Arch. Phys. Med. Rehabil.* 81 409–417. 10768528 10.1053/mr.2000.3839

[B12] Doherty C. Bleakley C. Hertel J. Caulfield B. Ryan J. Delahunt E. (2015). Lower extremity function during gait in participants with first time acute lateral ankle sprain compared to controls. *J. Electromyogr. Kinesiol.* 25 182–192. 25443172 10.1016/j.jelekin.2014.09.004

[B13] Doherty C. Bleakley C. Hertel J. Sweeney K. Caulfield B. Ryan J. (2014). Lower extremity coordination and symmetry patterns during a drop vertical jump task following acute ankle sprain. *Hum. Mov. Sci.* 38 34–46. 25240177 10.1016/j.humov.2014.08.002

[B14] Dubin J. C. Comeau D. McClelland R. I. Dubin R. A. Ferrel E. (2011). Lateral and syndesmotic ankle sprain injuries: a narrative literature review. *J. Chiropr. Med.* 10 204–219. 22014912 10.1016/j.jcm.2011.02.001 PMC3259913

[B15] Duncan G. H. Catherine Bushnell M. Lavigne G. J. (1989). Comparison of verbal and visual analogue scales for measuring the intensity and unpleasantness of experimental pain. *Pain* 37 295–303. 10.1016/0304-3959(89)90194-2 2755711

[B16] Duysens J. Tax A. A. Trippel M. Dietz V. (1992). Phase-dependent reversal of reflexly induced movements during human gait. *Exp. Brain Res.* 90 404–414. 10.1007/BF00227255 1397155

[B17] Dworkin R. H. Turk D. C. Revicki D. A. Harding G. Coyne K. S. Peirce-Sandner S. (2009). Development and initial validation of an expanded and revised version of the Short-form McGill Pain Questionnaire (SF-MPQ-2). *Pain* 144 35–42. 10.1016/j.pain.2009.02.007 19356853

[B18] Dworkin R. H. Turk D. C. Trudeau J. J. Benson C. Biondi D. M. Katz N. P. (2015). Validation of the Short-form McGill Pain Questionnaire-2 (SF-MPQ-2) in acute low back pain. *J. Pain* 16 357–366. 10.1016/j.jpain.2015.01.012 25640290

[B19] Elias L. J. Bryden M. P. Bulman-Fleming M. B. (1998). Footedness is a better predictor than is handedness of emotional lateralization. *Neuropsychologia* 36 37–43. 10.1016/s0028-3932(97)00107-3 9533385

[B20] Fortin K. Blanchette A. McFadyen B. J. Bouyer L. J. (2009). Effects of walking in a force field for varying durations on aftereffects and on next day performance. *Exp. Brain Res* 199 145–155. 10.1007/s00221-009-1989-9 19707747

[B21] Gallina A. Abboud J. Blouin J.-S. (2021). A task-relevant experimental pain model to target motor adaptation. *J. Physiol.* 599 2401–2417. 10.1113/JP281145 33638152

[B22] Graven-Nielsen T. Jansson Y. Segerdahl M. Kristensen J. D. Mense S. Arendt-Nielsen L. (2003). Experimental pain by ischaemic contractions compared with pain by intramuscular infusions of adenosine and hypertonic saline. *Eur. J. Pain* 7 93–102. 10.1016/s1090-3801(02)00069-1 12527322

[B23] Henriksen M. Rosager S. Aaboe J. Bliddal H. (2011). Adaptations in the gait pattern with experimental hamstring pain. *J. Electromyogr. Kinesiol.* 21 746–753. 10.1016/j.jelekin.2011.07.005 21824788

[B24] Hodges P. W. Tucker K. (2011). Moving differently in pain: a new theory to explain the adaptation to pain. *Pain* 152(3 Suppl): S90–S98. 10.1016/j.pain.2010.10.020 21087823

[B25] Ivins D. (2006). Acute ankle sprain: an update. *Am. Fam. Physician* 74 1714–1720. 17137000

[B26] Laskowski E. R. Newcomer-Aney K. Smith J. (2000). Proprioception. *Phys. Med. Rehabil.Clin. N. Am.* 11 323–340. 10810764

[B27] Levins A. D. Skinner H. B. Caiozzo V. J. (1998). Adaptive gait responses to plantar heel pain. *J. Rehabil. Res. Dev.* 35 289–293. 9704312

[B28] Madeleine P. Voigt M. Arendt-Nielsen L. (1999). Reorganisation of human step initiation during acute experimental muscle pain. *Gait Posture.* 10 240–247. 10.1016/s0966-6362(99)00036-3 10567756

[B29] Moseley G. L. Hodges P. W. (2005). Are the changes in postural control associated with low back pain caused by pain interference? *Clin. J. Pain* 21 323–329. 10.1097/01.ajp.0000131414.84596.99 15951650

[B30] Moseley G. L. Nicholas M. K. Hodges P. W. (2004). Does anticipation of back pain predispose to back trouble? *Brain* 127 2339–2347. 10.1093/brain/awh248 15282214

[B31] Nielsen J. B. (2003). How we walk: central control of muscle activity during human walking. *Neuroscientist* 9 195–204. 10.1177/1073858403009003012 15065815

[B32] O’Connor S. R. Bleakley C. M. Tully M. A. McDonough S. M. (2013). Predicting functional recovery after acute ankle sprain. *PLoS One* 8:e72124. 10.1371/journal.pone.0072124 23940806 PMC3734311

[B33] Petersen K. L. Rowbotham M. C. (1999). A new human experimental pain model: the heat/capsaicin sensitization model. *Neuroreport* 10 1511–1516. 10.1097/00001756-199905140-00022 10380972

[B34] Punt I. M. Ziltener J.-L. Laidet M. Armand S. Allet L. (2015). Gait and physical impairments in patients with acute ankle sprains who did not receive physical therapy. *PM R* 7 34–41. 10.1016/j.pmrj.2014.06.014 24998405

[B35] Reading A. E. (1982). A comparison of the McGill Pain Questionnaire in chronic and acute pain. *Pain* 13 185–192. 10.1016/0304-3959(82)90028-8 6889722

[B36] Seeley M. K. Park J. King D. Hopkins J. T. A. (2013). Novel experimental knee-pain model affects perceived pain and movement biomechanics. *J. Athl. Train.* 48 337–345. 10.4085/1062-6050-48.2.02 23675793 PMC3655747

[B37] Sinatra R. (2010). Causes and consequences of inadequate management of acute pain. *Pain Med.* 11 1859–1871. 10.1111/j.1526-4637.2010.00983.x 21040438

[B38] Smith S. A. Micklewright D. Winter S. L. Mauger A. R. (2020). Muscle pain induced by hypertonic saline in the knee extensors decreases single-limb isometric time to task failure. *Eur. J. Appl. Physiol.* 120 2047–2058. 10.1007/s00421-020-04425-2 32613451 PMC7419372

[B39] Spaich E. G. Arendt-Nielsen L. Andersen O. K. (2004). Modulation of lower limb withdrawal reflexes during gait: a topographical study. *J. Neurophysiol.* 91 258–266. 10.1152/jn.00360.2003 12968008

[B40] Sterling M. Jull G. Wright A. (2001). The effect of musculoskeletal pain on motor activity and control. *J. Pain.* 2 135–145. 10.1054/jpai.2001.19951 14622823

[B41] Stohler C. S. Zhang X. Lund J. P. (1996). The effect of experimental jaw muscle pain on postural muscle activity. *Pain* 66 215–221. 10.1016/0304-3959(96)03026-6 8880843

[B42] Tucker K. Larsson A.-K. Oknelid S. Hodges P. (2012). Similar alteration of motor unit recruitment strategies during the anticipation and experience of pain. *Pain* 153 636–643. 10.1016/j.pain.2011.11.024 22209423

