# Supplementary figures and images for: Gait Adaptation to a Phase-Specific Nociceptive Electrical Stimulation Applied at the Ankle: A Model to Study Musculoskeletal-Like Pain
Source: Front Hum Neurosci. 2021 Dec 17;15:762450. doi: 10.3389/fnhum.2021.762450 (PMC8718644; doi:10.3389/fnhum.2021.762450)

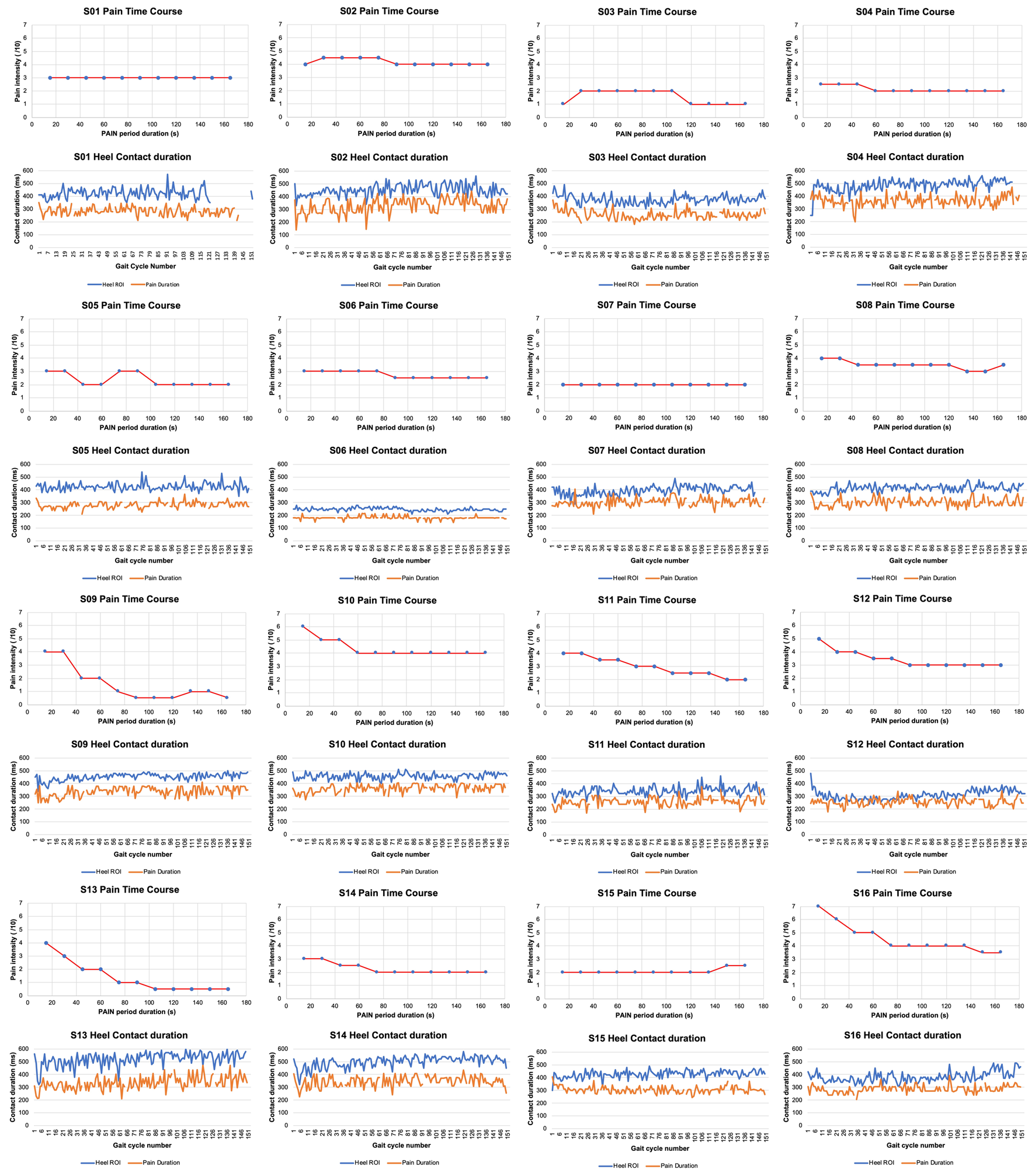

Supplement: Supplementary Figure 1 — Individual time courses for the pain intensity (red line), pressure duration (pressure-sensitive insoles; blue line), and foot switch located under the right heel (orange line) for each participant. [file Image_1.tif]
